# Down regulation of miR-143 is related with tumor size, lymph node metastasis and HPV16 infection in cervical squamous cancer

**DOI:** 10.1186/1746-1596-9-88

**Published:** 2014-04-28

**Authors:** Yanxia Chen, Cailing Ma, Wei Zhang, Zhifang Chen, Li Ma

**Affiliations:** 1 Department of Gynecology, the First Affiliated Hospital of Xinjiang Medical University, No. 137 Liyushan South Road, Urumqi, Xinjiang 830054, P.R. China; 2 Department of Pathology, the First Affiliated Hospital of Xinjiang Medical University, Urumqi, Xinjiang 830054, P.R. China

## Abstract

**Objective:**

This study is to investigate the role of miR-143 expression in cervical squamous cell carcinoma (SCC).

**Methods:**

The expression level of miR-143 was examined by quantitative real-time PCR. Human papillomavirus (HPV) genotype was detected by HPV genotype detection kit. The expression level of bcl-2 was detected by immunohistochemistry.

**Results:**

The positive rate of HPV was 78% in the patients of cervical SCC. The most prevalent genotype was HPV16, with a positive rate of 42%. The expression level of miR-143 was significantly lower in the cervical SCC tissues than that in the normal cervical tissues (Z = −2.180, P = 0.029). Down-regulated miR-143 expression was associated with tumor size, lymph node metastasis and HPV16 infection in cervical cancer patients. No significant associations were found between the expression levels of miR-143 and age, clinical stage, differentiation or lymph vascular space invasion. And, in cervical SCC patients after treatment with Taxol chemotherapy, the expression level of miR-143 was higher and the positive expression of bcl-2 protein was lower. However, the differences in expression changes of miR-143 and bcl-2 were not statistically significant (miR-143, Z = −0.763, P = 0.446; bcl-2 protein, χ^2^ = 2.277, P = 0.131).

**Conclusion:**

Down-regulated miR-143 is related with tumor size, lymph node metastasis and HPV16 infection in cervical SCC, but miR-143 does not participate in the Taxol sensitivity response.

**Virtual slides:**

The virtual slide(s) for this article can be found here: http://www.diagnosticpathology.diagnomx.eu/vs/1401279451112150.

## Introduction

Cervical cancer is caused by the activation of oncogenes and the inactivation of tumor suppressor genes, which are induced by carcinogens through different mechanisms. Persistent infection of the human papillomavirus (HPV) is an important factor causing cervical cancer. Genes of E6 and E7 of HPV are able to inactivate the cellular tumor suppressor genes and induce the over expression of the anti-apoptotic genes. Besides HPV, factors from host cells are also critical in the process of cervical malignant transformation. MicroRNAs (miRNAs) are a class of small, endogenous, single-stranded, non-coding RNA molecules, which can regulate cell proliferation, differentiation and apoptosis through targeting specific genes. Thus they might be involved in carcinogenesis or tumor suppression. Abnormal expression of miR-143 is observed in many types of tumors, and it is involved in the tumor responses to chemotherapy [1]. Bcl-2 is the major target of miR-143. It is reported that HPV infection may alter miRNA profiles in squamous cell carcinoma of the head and neck cell lines [2]. Lajer et al. found that the miR-143/miR-145 may be involved in the pathogenesis of HPV-related head and neck cancer and cervical cancer [3]. Wang et al. showed that miR-143 and miR-145 expression was down-regulated in cervical cancer tissues and HPV-infected raft tissues with pre-neoplastic lesions. Overexpression of miR-143 and miR-145 suppressed HeLa cell growth. Their findings suggest that down regulation of miR-143 and miR-145 may favor cell growth of cervical cancer [4]. Georgios et al. found that miR-143 expression did not correlate with histology of cervical cancer [5]. Therefore, miR-143 plays an important role in cervical cancer.

Neoadjuvant chemotherapy (NACT) provides surgical opportunities for some patients, with enhanced efficacy of surgery and radiotherapy [6]. It is reported that miRNAs are involved in drug resistance of cancer cells. For example, Zhang et al. studied the miRNA signature in stage II colon cancer using miRNA microarrays. They found that six miRNAs, including miR-21-5p, miR-20a-5p, miR-103a-3p, miR-106b-5p, miR-143-5p, and miR-215, might be able to predict which patients benefit from adjuvant chemotherapy [7]. Borralho et al. reported that miR-143 increased the sensitivity of HCT116 human colorectal cancer cells to 5-fluorouracil, probably through extracellular-regulated protein kinase 5/nuclear factor-kappaB regulated pathways [8]. Xu et al. found that miR-143 enhanced the sensitivity of prostate cancer cells to docetaxel [9]. However, little is known about the role of miR-143 in modulating the cervical cancer cell response to Taxol.

The incidence rate of cervical cancer among the Uighur in the Xinjiang Uygur Autonomous Region is three to four times of that in Han race. And there are more cases in the Xinjiang Uygur Autonomous Region with advanced stages of cervical cancer. This study aims to investigate the role of miR-143 expression in cervical SCC through detecting miR-143 expression, analyzing the relationship between miR-143 expression and clinical pathological features of cervical cancer, and assessing the sensitivity of miR-143 and bcl-2 expression to Taxol treatment. Therefore, in this study, the miR-143 expression levels in cervical tumors infected with different HPV genotypes and in the cervical cancer tissues before and after NACT treatment were detected. Then the relationship between the miR-143 and the clinical and pathological features of cervical squamous cell carcinoma (SCC) in Uighur was analyzed. Additionally, the expression of miR-143 and its target protein bcl-2 was also assessed.

## Materials and methods

### Clinical data

Paraffin-embedded cervical tissues from 97 patients were collected in this study. The 97 patients were hospitalized from June 2010 to January 2012 in First Teaching Hospital of Xinjiang Medical University. Among them, 20 control patients had normal cervixes and 77 patients had cervical SCC. Patients with cervical SCC were newly diagnosed SCC cases, and did not receive radiotherapy or chemotherapy before surgery. The clinical pathological data of the 77 cervical SCC patients were shown in Table 1. The clinical data for 20 control patients was shown in Table 2. Exfoliated cervical cells were collected and the genotypes of HPV were classified by blind detection. Among the patients with cervical SCC, there were 59 patients who underwent radical hysterectomy and pelvic lymphadenectomy, and 18 patients who underwent cervical biopsy. And there were 16 cases at stage I, 43 cases at stage II, and 18 cases at stage III-IV; 27 well differentiated cases and 50 moderate and poor differentiated cases; 23 patients with tumor diameters > 4 cm, and 54 patients with tumor diameters ≤ 4 cm; 43 cases without lymph node metastasis, and 16 cases with lymph node metastasis; 13 cases of lymph vascular space invasion (LVSI), and 46 cases without LVSI. The normal cervical tissues were collected from patients who underwent uterus hysterectomy because of myoma. Ages of the patients with normal cervixes ranged from 30 to 76 years old, with the mean age of 45 years old; and those of the cervical SCC patients ranged from 27 to 80 years old, with the mean age of 47 years old. There was no statistical significance in age between patients with normal cervical tissues and patients with cervical SCC.

**Table 1:** Clinical pathological data of cervical SCC patients (n = 77)

**Cervical SCC patients**	**Age (year)**	**Selection date**	**Clinical stage**	**Differentai-ation degree**	**Lymph node metastasis**	**Tumor size (cm)**	**LVSI**	**HPV infection**
1	46	2010.7.2	Stage I	Well differentiation	No	≤ 4	No	Single/multiple HPV16 infection
2	44	2010.7.5	Stage II	Moderate and poor differentiation	No	≤ 4	No	Single/multiple HPV16 infection
3	35	2010.7.13	Stage I	Well differentiation	No	≤ 4	Yes	Negative
4	39	2010.7.26	Stage II	Moderate and poor differentiation	No	≤ 4	No	Negative
5	38	2010.7.30	Stage I	Moderate and poor differentiation	Yes	> 4	No	Other HPV type
6	30	2010.8.2	Stage I	Moderate and poor differentiation	No	≤ 4	No	Single/multiple HPV16 infection
7	47	2010.8.5	Stage III-IV	Well differentiation	Yes	≤ 4	No	Single/multiple HPV16 infection
8	41	2010.8.12	Stage II	Well differentiation	No	> 4	No	Single/multiple HPV16 infection
9	44	2010.8.23	Stage II	Moderate and poor differentiation	No	≤ 4	No	Negative
10	38	2010.8.23	Stage III-IV	Moderate and poor differentiation	No	≤ 4	No	Other HPV type
11	27	2010.8.25	Stage III-IV	Well differentiation	No	≤ 4	No	Other HPV type
12	48	2010.8.30	Stage I	Moderate and poor differentiation	No	≤ 4	No	Negative
13	42	2010.9.7	Stage II	Well differentiation	Yes	> 4	Yes	Single/multiple HPV16 infection
14	44	2010.9.10	Stage I	Moderate and poor differentiation	No	≤ 4	No	Negative
15	42	2010.9.17	Stage I	Moderate and poor differentiation	No	> 4	No	Single/multiple HPV16 infection
16	45	2010.9.27	Stage II	Moderate and poor differentiation	No	> 4	No	Single/multiple HPV16 infection
17	44	2010.9.28	Stage II	Moderate and poor differentiation	No	1	No	Single/multiple HPV16 infection
18	42	2010.9.28	Stage II	Moderate and poor differentiation	No	≤ 4	No	Other HPV type
19	47	2010.9.30	Stage II	Moderate and poor differentiation	Yes	> 4	No	Single/multiple HPV16 infection
20	39	2010.10.18	Stage I	Moderate and poor differentiation	No	≤ 4	No	Single/multiple HPV16 infection
21	48	2010.10.28	Stage II	Well differentiation	No	≤ 4	No	Single/multiple HPV16 infection
22	36	2010.11.2	Stage II	Moderate and poor differentiation	Yes	> 4	Yes	Other HPV type
23	43	2010.11.16	Stage II	Moderate and poor differentiation	No	≤ 4	No	Other HPV type
24	34	2010.11.24	Stage I	Moderate and poor differentiation	No	≤ 4	No	Other HPV type
25	46	2010.12.10	Stage III-IV	Well differentiation	---	> 4	---	Other HPV type
26	45	2010.12.16	Stage II	Well differentiation	---	≤ 4	---	Other HPV type
27	32	2010.12.27	Stage III-IV	Well differentiation	---	≤ 4	---	Single/multiple HPV16 infection
28	27	2011.1.7	Stage III-IV	Well differentiation	---	> 4	---	Single/multiple HPV16 infection
29	40	2011.1.14	Stage III-IV	Well differentiation	---	≤ 4	---	Single/multiple HPV16 infection
30	29	2011.1.17	Stage III-IV	Well differentiation	---	≤ 4	---	Other HPV type
31	42	2011.2.21	Stage I	Well differentiation	---	≤ 4	---	Single/multiple HPV16 infection
32	35	2011.2.25	Stage I	Well differentiation	---	≤ 4	---	Negative
33	29	2011.3.1	Stage III-IV	Well differentiation	---	> 4	---	Negative
34	38	2011.3.10	Stage III-IV	Well differentiation	---	≤ 4	---	Other HPV type
35	41	2011.3.15	Stage III-IV	Well differentiation	---	≤ 4	---	Single/multiple HPV16 infection
36	28	2011.3.19	Stage II	Moderate and poor differentiation	---	≤ 4	---	Single/multiple HPV16 infection
37	40	2011.3.21	Stage II	Moderate and poor differentiation	---	> 4	---	Negative
38	37	2011.4.9	Stage II	Moderate and poor differentiation	---	≤ 4	---	Other HPV type
39	43	2011.4.13	Stage II	Moderate and poor differentiation	---	> 4	---	Single/multiple HPV16 infection
40	29	2011.4.18	Stage II	Well differentiation	---	> 4	---	Other HPV type
41	43	2011.4.21	Stage II	Moderate and poor differentiation	---	> 4	---	Single/multiple HPV16 infection
42	53	2011.4.22	Stage III-IV	Moderate and poor differentiation	No	> 4	Yes	Negative
43	66	2011.5.10	Stage III-IV	Moderate and poor differentiation	No	≤ 4	No	Other HPV type
44	54	2011.5.16	Stage III-IV	Moderate and poor differentiation	No	≤ 4	No	Other HPV type
45	56	2011.5.23	Stage II	Moderate and poor differentiation	No	≤ 4	No	Other HPV type
46	57	2011.5.25	Stage II	Moderate and poor differentiation	Yes	≤ 4	Yes	Other HPV type
47	54	2011.5.30	Stage II	Well differentiation	No	≤ 4	No	Other HPV type
48	54	2011.6.3	Stage III-IV	Moderate and poor differentiation	Yes	≤ 4	No	Other HPV type
49	66	2011.6.10	Stage II	Moderate and poor differentiation	Yes	≤ 4	No	Other HPV type
50	77	2011.6.14	Stage III-IV	Moderate and poor differentiation	Yes	≤ 4	No	Single/multiple HPV16 infection
51	50	2011.6.15	Stage II	Well differentiation	No	≤ 4	No	Single/multiple HPV16 infection
52	68	2011.6.20	Stage II	Well differentiation	No	≤ 4	No	Negative
53	61	2011.7.11	Stage II	Moderate and poor differentiation	No	≤ 4	Yes	Negative
54	69	2011.7.15	Stage II	Moderate and poor differentiation	No	≤ 4	Yes	Other HPV type
55	49	2011.7.18	Stage II	Moderate and poor differentiation	No	> 4	No	Single/multiple HPV16 infection
56	49	2011.7.18	Stage II	Moderate and poor differentiation	Yes	> 4	Yes	Negative
57	54	2011.8.1	Stage II	Moderate and poor differentiation	No	≤ 4	No	Single/multiple HPV16 infection
58	51	2011.8.25	Stage III-IV	Well differentiation	No	≤ 4	No	Single/multiple HPV16 infection
59	56	2011.8.26	Stage II	Moderate and poor differentiation	Yes	≤ 4	Yes	Single/multiple HPV16 infection
60	53	2011.913	Stage II	Moderate and poor differentiation	No	> 4	No	Single/multiple HPV16 infection
61	50	2011.9.16	Stage I	Well differentiation	No	≤ 4	No	Other HPV type
62	51	2011.9.21	Stage II	Moderate and poor differentiation	No	≤ 4	No	Single/multiple HPV16 infection
63	49	2011.9.23	Stage I	Well differentiation	No	≤ 4	No	Negative
64	57	2011.10.14	Stage II	Moderate and poor differentiation	No	≤ 4	Yes	Negative
65	61	2011.11.2	Stage II	Well differentiation	No	> 4	No	Other HPV type
66	52	2011.11.15	Stage II	Moderate and poor differentiation	No	≤ 4	No	Negative
67	50	2011.11.17	Stage II	Moderate and poor differentiation	Yes	> 4	No	Single/multiple HPV16 infection
68	56	2011.11.21	Stage I	Moderate and poor differentiation	No	≤ 4	Yes	Negative
69	64	2011.11.25	Stage I	Moderate and poor differentiation	No	≤ 4	No	Single/multiple HPV16 infection
70	80	2011.12.2	Stage II	Moderate and poor differentiation	No	≤ 4	Yes	Single/multiple HPV16 infection
71	61	2011.12.2	Stage II	Well differentiation	Yes	≤ 4	No	Other HPV type
72	49	2011.12.5	Stage II	Moderate and poor differentiation	No	> 4	No	Other HPV type
73	49	2011.12.16	Stage II	Moderate and poor differentiation	Yes	≤ 4	No	Other HPV type
74	59	2012.1.10	Stage II	Moderate and poor differentiation	Yes	≤ 4	No	Other HPV type
75	50	2012.1.13	Stage II	Moderate and poor differentiation	Yes	> 4	No	Single/multiple HPV16 infection
76	67	2012.1.16	Stage I	Moderate and poor differentiation	No	≤ 4	Yes	Other HPV type
77	58	2012.1.19	Stage III-IV	Moderate and poor differentiation	---	≤ 4	---	Negative

Note: SCC, squamous cell carcinoma; LVSI, lymph vascular space invasion. “---” indicates that no such examination was performed.

Among the 77 cervical patients, 59 cases received extensive hysterectomy and pelvic lymphadenectomy and 18 cases were not treated with surgery. Lymph node metastasis and LVSI were not examined in these 18 cases who were not treated with surgery.

**Table 2:** Clinical data of 20 control patients with normal cervixes (n = 20)

**Patients**	**Age (year)**	**Selection date**	**Patients**	**Age (year)**	**Selection date**
1	43	2010.7.14	11	42	2011.5.13
2	32	2010.7.29	12	40	2011.6.16
3	50	2010.8.13	13	39	2011.7.15
4	30	2010.9.15	14	44	2011.8.16
5	37	2010.9.16	15	66	2011.9.13
6	41	2010.10.19	16	31	2011.9.16
7	40	2010.12.17	17	45	2011.10.18
8	47	2011.1.17	18	76	2011.11.21
9	59	2011.3.17	19	42	2011.12.2
10	63	2011.4.20	20	50	2012.1.10

Fresh cervical tissues from another 34 cervical SCC patients who were hospitalized in the hospital from January 2012 to December 2012 for pre-surgical chemotherapy were also selected. They were diagnosed as stage I b2 – II b cervical SCC by at least two co-chief physicians. They were treated with NACT before surgery. Clinical data of 34 cervical SCC patients who received NACT before surgery were shown in Table 3. A matched pair of tissue samples from the same patient prior to chemotherapy and after chemotherapy was collected from all the 34 patients. Each sample was divided into two parts: one part was put into liquid nitrogen within 10 minutes after separation, and then stored at −80°C, and the other part was used for pathological diagnosis after hematoxylin-eosin (HE) staining. The ages of cervical SCC patients with chemotherapy ranged from 32 to 67 years old, with a mean age of 47.5 years old.

**Table 3:** Clinical data of 34 cervical SCC patients who received NACT before surgery (n = 34)

**Patients**	**Age (year)**	**Selection date**	**Clinical stage**	**NACT efficacy**
1	45	2012.1.27	Stage I_b2_	Effective
2	34	2012.2.4	Stage IIa	Effective
3	36	2012.3.12	Stage IIb	Effective
4	47	2012.4.15	Stage I_b2_	Not effective
5	57	2012.4.17	Stage I_b2_	Effective
6	39	2012.5.15	Stage IIa	Effective
7	45	2012.5.18	Stage IIa	Effective
8	42	2012.6.3	Stage IIb	Not effective
9	32	2012.6.17	Stage I_b2_	Effective
10	66	2012.6.26	Stage IIa	Not effective
11	59	2012.7.14	Stage IIb	Not effective
12	46	2012.8.4	Stage I_b2_	Effective
13	49	2012.8.15	Stage IIa	Not effective
14	51	2012.8.24	Stage I_b2_	Effective
15	35	2012.8.29	Stage I_b2_	Effective
16	38	2012.9.16	Stage IIa	Not effective
17	40	2012.9.19	Stage I_b2_	Effective
18	43	2012.10.7	Stage IIa	Effective
19	58	2012.10.11	Stage IIb	Effective
20	63	2012.10.17	Stage IIa	Not effective
21	53	2012.10.19	Stage I_b2_	Effective
22	48	2012.10.25	Stage I_b2_	Effective
23	43	2012.10.28	Stage IIa	Effective
24	45	2012.11.1	Stage IIb	Not effective
25	44	2012.11.5	Stage IIa	Effective
26	61	2012.11.16	Stage I_b2_	Not effective
27	39	2012.11.23	Stage IIa	Effective
28	67	2012.11.25	Stage IIa	Effective
29	56	2012.11.30	Stage I_b2_	Effective
30	44	2012.12.2	Stage I_b2_	Effective
31	33	2012.12.10	Stage IIa	Not effective
32	54	2012.12.15	Stage I_b2_	Effective
33	57	2012.12.19	Stage IIa	Effective
34	49	2012.12.22	Stage I_b2_	Effective

Note: SCC, squamous cell carcinoma. NACT, neoadjuvant chemotherapy.

The informed consents were obtained from all patients, and the study was approved by the ethics committee of Xinjiang Medical University. Female patients with other malignant tumors, gestation, breast-feeding, or complications who were intolerant to chemotherapy were excluded. Clinical staging was performed according to the 2009 International Federation of Gynecology and Obstetrics (FIGO) staging criteria, and the images were read by two pathology experts independently based on the WHO histopathological criteria.

### Reagents

RNeasy FFPE Kit (cat. no. 73504) was purchased from QIAGEN Ltd (Hilden, Germany). Trizol was obtained from Invitrogen CO., Ltd. (Carlsbad, California, USA). Reverse transcription Kit (#K1622) and real-time fluorescent quantitative PCR Mix (#K0221) were purchased from Thermo Fisher Scientific Inc (Vilnius, Lithuania). The specific reverse transcriptase and PCR primers of U6 and miR-143 were synthesized by Taihe Biotechnology Co., Ltd. (Beijing, China). The bcl-2 antibody (sc-7382) was purchased from Santa Cruz Biotechnology Inc (California, USA). The universal secondary antibody (PV-6000), goat serum and diaminobenzidine (DAB) reagent were all purchased from Beijing Zhong Shan-Golden Bridge Biological Technology CO., Ltd. (Beijing, China). Cell lysis buffer and the HPV genotype detection kit were products from Chaozhou Kaipu Biochemistry Co. Ltd. (Chaozhou, China). ABI7500 real-time PCR system was purchased from Applied Biosystems, Inc. (New York, USA), and the OLYMPUS microscopy was from Olympus optical Co., Ltd. (Tokyo, Japan).

### HPV genotype detection

Exfoliated cervical cell specimens were obtained by cervical brush clockwise rotation for five turns at the cervix. DNA was extracted from these specimens for determination of HPV genotypes. For DNA amplication, 1 μl of DNA and 24 μl of HPV PCR reaction solution (containing Taq polymerase, dNTP and primers) were mixed together to form a 25 μl system. Then PCR products were hybridized to films according to the manufacturer’s instruction. During the color development, 0.5 ml of alkaline phosphatase chromogenic substrates were added and incubated for 2–5 minutes at 36°C. Results were analyzed within 1 hour after color development through visual observation. Positive points were defined as clear visible blue purple dots. There were one PCR reaction control and one hybridization coloration quality control point on each film. The HPV genotypes were determined based on their distribution sites on the films, including 21 HPV genotypes of 6, 11, 16, 18, 31, 33, 35, 39, 42, 43, 44, 45, 51, 52, 53, 56, 58, 59, 66, 68 and 81 (CP8304).

### Reverse transcription and real-time PCR

Total RNAs of paraffin-embedded cervical tissues were extracted according to the RNeasy FFPE kit’s instruction, while those of the fresh cervical tissues were extracted by Trizol. U6 was used as internal standard control. The volume of reverse transcription reaction system was 20 μl, containing 200 ng of total RNAs, 1 μl of reverse transcription primers diluted to 12 μl with water, 4 μl of buffer, 1 μl of RNase inhibitor, 2 μl of dNTP and 1 μl of M-MuLV reverse transcriptase. Reverse transcriptions were performed. The miR-143-specific primer sequence for reverse transcription was: 5’-GTCGTATCCAGTGCGTGTCGTGGAGTCGGCAATTGCACTGGATACGACTGAGCTA-3’. The miR-143-specific PCR primer sequences for real-time PCR were: forward, 5’-AGTGCGTGTCGTGGAGT-3’; reverse, 5’-GCCTGAGATGAAGCACTGT-3’. The U6 PCR primer sequences were: forward, 5’-CTCGCTTCGGCAGCACA-3’; reverse, 5’-ACGCTTCACGAATTTTGCGT-3’.

Each sample was repeated 3 times. The PCR products were analyzed by 2.5% agarose gel electrophoresis. The number of cycles for each reaction tube to reach the pre-established fluorescence threshold was recorded as Ct value, and the relative expression quantities of miR-143 were presented as N = 2^-ΔΔCt^. For the comparison of cervical SCC group to normal cervix group, ΔΔCt = (Ct_miR-143_-Ct_U6_)_SCC_ - (average Ct_miR-143_ - average Ct_U6_)_normal_; For the comparison of cervical SCC group after chemotherapy to the cervical SCC group before chemotherapy, ΔΔCt = (Ct_miR-143_ - Ct_U6_)_after_ - (Ct_miR-143_ - Ct_U6_)_before_.

### Immunohistochemistry

Immunohistochemistry was performed in fresh cervical tissues to detect the expression of Bcl-2. Briefly, slices (4 μm in thickness) were incubated in 3% H_2_O_2_ for 10 minutes at room temperature to block endogenous peroxidase. Bcl-2 mouse monoclonal antibody (1: 200) was added and kept at 4°C overnight, and then incubated for 1 hour at room temperature. Then, the universal IgG antibody conjugated with HRP was added and incubated at 37°C for 25 minutes. DAB solution was used for color development, and the staining results were observed under microscopy. Finally, the slices were counterstained with hematoxylin for 10 minutes, differentiated with hydrochloric acid ethanol, and then mounted with neutral gum after dehydration. The slices were observed under microscopy. Bcl-2 positive breast cancer tissue was used as bcl-2 positive control and PBS instead of the primary antibody was used as negative control. Positive staining cells were stained brown-yellow or yellow in the cytoplasm or cell membrane. Five fields were randomly selected on each slice and 200 cells were counted in each field. Then the percentage of positive cells in all counted cells was calculated. Negative staining was defined as no positive staining on the whole slice or the percentage of positive cells < 10% and positive staining was defined as the percentage of positive cells > 10%.

### NACT

The chemotherapy regimen included Taxol (175 mg/m^2^) and carboplatin (dosage of which was calculated based on the age, serum creatinine and weight of the patient). Taxol and carboplatin were administered within one day. Two courses of treatments were taken with an interval of 3 weeks, and surgery was performed 2 – 3 weeks after the chemotherapy. The tumor sizes of the patients treated with NACT were measured through gynecological and imaging examinations before chemotherapy, and measured by pathological examinations after surgery. According to the WHO criteria, effectiveness of treatment was defined as the follows: complete remission, tumor is completely disappeared; partial remission, the tumor size decreases more than 50%; stable or no change, the tumor size increases or decreases no more than 25%, and no new lesions appear; progression, new lesions appear or the size of tumor increases more than 25% during the treatment. Complete and partial remissions were defined as effective.

### Statistical analysis

Data were processed using SPSS 17.0 software. The data was non-normal distribution and thus median and quartile were used. Mann–Whitney U test was used for comparison between two groups, while Kruskal-Wallis H test was used for comparison among multiple groups. P < 0.05 was considered as statistically significant.

## Results

### The miR-143 expression in normal cervix and cervical SCC tissues and its correlation with clinical pathological features of cervical SCC

**Figure 1:**
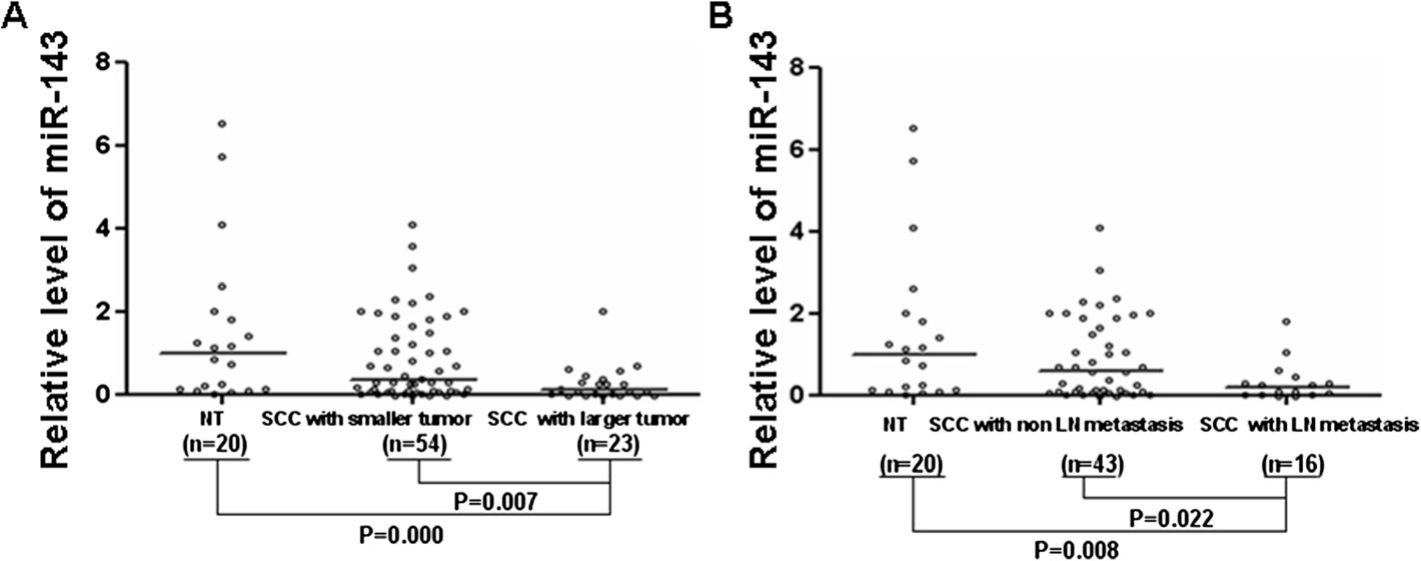
**Comparison of miR-143 expression in normal cervical tissue and cervical SCC tissue with different tumor size and lymph node metastasis.** Real time PCR was conducted to detect miR-143 expression. Relative expression of miR-143 was calculated using the 2^-ΔΔCt^ method. **(A)** Comparison of miR-143 expression in normal cervical tissue (NT) and cervical SCC tissue with smaller tumor (tumor diameter ≤ 4 cm) and larger tumor (tumor diameter > 4 cm). **(B)** Comparison of miR-143 expression in normal cervical tissue (NT) and cervical SCC tissue with or without lymph node (LN) metastasis.

To determine the expression levels of miR-143 in normal cervix and cervical SCC, real time PCR was performed. Quantitative results of miR-143 expression and its correlation with clinical pathological features of cervical SCC were shown in Figure 1 and Table 4. In comparison with normal cervix, the expression level of miR-143 in cervical SCC group was decreased, and the difference was statistically significant (Z = −2.180, P = 0.029). The expression of miR-143 in cervical SCC with tumor diameter ≤ 4 cm was significantly higher than that with tumor diameter > 4 cm (Z = −2.705, P = 0.007) (Table 4 and Figure 1A). And, miR-143 expression level in cervical SCC without lymph node metastasis was higher than that with lymph node metastasis and the difference was also statistically significant (Z = −2.293, P = 0.022) (Table 4 and Figure 1B).

**Table 4:** Correlation between miR-143 expression and cervical SCC clinical pathological features

**Clinical characters**	**Cases (n)**	**miR-143 expression**	**Z**	**P**
**Cervical SCC**			−2.180	0.029*
No	20	1.000 (0.241-1.878)		
Yes	77	0.280 (0.059-1.050)		
**Age (years old)**			−1.236	0.217
≤ 50	41	0.259 (0.143-0.707)		
> 50	36	0.337 (0.167-1.092)		
**Clinical Stage**			2.673	0.263
I	16	0.387 (0.176-1.258)		
II	43	0.268 (0.131-0.698)		
III-IV	18	0.287 (0.155-0.905)		
**Pathological Grade**			−1.142	0.253
Well differentiation	27	0.320 (0.198-0.859)		
Moderate and poor differentiation	50	0.258 (0.118-1.052)		
**Lymph node metastasis**			−2.293	0.022*
No	43	0.578 (0.209-1.384)		
Yes	16	0.175 (0.103-0.544)		
**Tumor diameter**			−2.705	0.007*
≤ 4 cm (smaller tumor)	54	0.345 (0.112-1.490)		
> 4 cm (larger tumor)	23	0.125 (0.103-0.459)		
**LVSI**			−0.567	0.571
No	46	0.294 (0.159-1.075)		
Yes	13	0.607 (0.293-1.501)		
**HPV infection**			9.050	0.011*
HPV negative	17	1.053 (0.289-1.412)		
HPV16 infection (single or multiple)^△^	32	0.230 (0.105-0.496)	−2.647	0.008*
Other genotype HPV infection^△^	28	0.524 (0.109-1.233)	−1.651	0.099

Note: *P < 0.05. ^△^in comparison with HPV negative group.

SCC, squamous cell carcinoma; LVSI, lymph vascular space invasion.

In addition, the HPV genotype was detected. In cervical SCC patients, there were 60 HPV positive patients (78%). Among them, 32 were infected by HPV16 (42%), including 25 single infections (33%) and 7 mixed infections (9%), and 28 patients were infected by other HPV genotypes, in which HPV18, HPV52 and HPV58 were the major genotypes. Statistically, the expression of miR-143 in HPV negative cervical SCC patients was significantly higher than that in HPV positive cervical SCC patients (Z =9.050, P = 0.011) (Table 4). Meanwhile, in comparison with HPV negative cervical SCC patients, the expression of miR-143 in the HPV16 positive cervical SCC patients was significantly lower (Z = −2.647, P = 0.008) whereas in cervical SCC patients infected with other HPV genotypes, the difference was not significant (Z = −1.651, P = 0.099). However, the miR-143 expression levels between ages, clinical stages, pathological grades and LVSI were not significantly different.

Collectively, these data suggest that miR-143 expression in cervical SCC patients was down-regulated and that it was significantly correlated with lymph node metastasis, tumor diameter and HPV infection, but not with ages, clinical stages, pathological grades or LVSI.

### Evaluation of miR-143 expression as predictor of tumor size and lymph node metastasis

**Figure 2:**
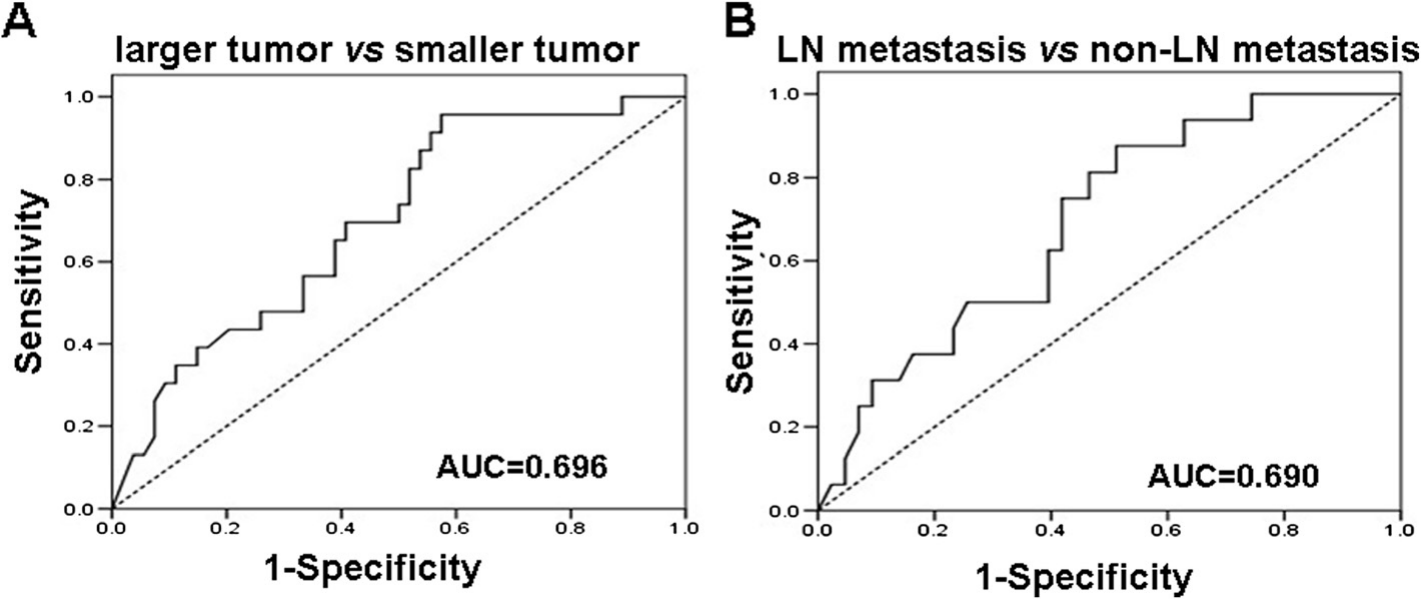
**Analysis of miR-143 expression in cervical SCC patients by ROC curve. (A)** ROC curve of miR-143 expression in cervical SCC patients with different tumor diameter. Tumor size with tumor diameter > 4 cm was defined as larger tumor and ≤ 4 cm as smaller tumor. **(B)** ROC curve of miR-143 expression in cervical SCC patients with and without lymph node (LN) metastasis.

The specificity and sensitivity of miR-143 evaluation on tumor size and lymph node metastasis of cervical SCC were analyzed by receiver operating characteristic (ROC) curve (Figure 2). Tumor size with tumor diameter > 4 cm was defined as larger tumor and ≤ 4 cm as smaller tumor. As shown in Figure 2A, the AUC of miR-143 to differentiate larger tumor from smaller tumor was 0.696 (95% confidence interval: 0.573 - 0.818, P = 0.007). If the relative expression level of 0.286 was used as the evaluation critical value, the sensitivity and specificity of miR-143 to differentiate tumor size were 70.5% and 61.0%, respectively. As shown in Figure 2B, the AUC of miR-143 to evaluate whether there was lymph node metastasis was 0.690 (95% confidence interval: 0.555 - 0.836, P = 0.022). If the relative expression value of 0.314 was used as the evaluation critical value, the sensitivity and specificity of miR-143 to differentiate whether there was lymph node metastasis were 75.0% and 60.0%, respectively. Thus miR-143 may be used as an indicator for the evaluation of tumor size and lymph node metastasis of cervical SCC, with good specificity and sensitivity.

### Expression of miR-143 and its target gene bcl-2 in cervical SCC before and after NACT

**Figure 3:**
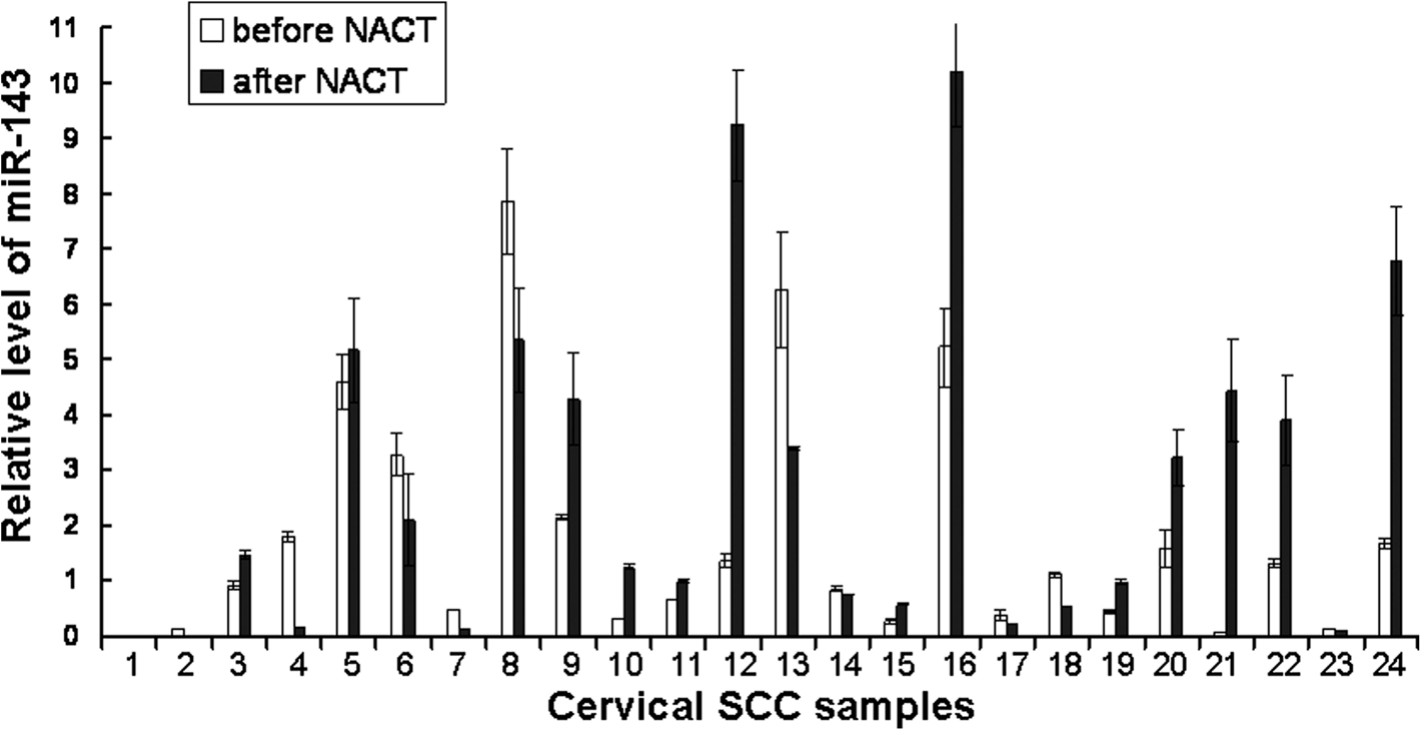
**Comparison of the relative miR-143 expression level in cervical SCC tissues before and after neoadjuvant chemotherapy (NACT).** Cervical SCC tissues were collected from 24 cervical SCC patients with effective NACT. Real time PCR was performed to detect miR-143 expression before and after NACT. Relative expression of miR-143 was calculated using the 2^-ΔΔCt^ method.

As mentioned in the Materials and methods, Taxol was used in NACT. To investigate whether miR-143 participated in the Taxol sensitivity response, we further analyzed expression of miR-143 and its target gene bcl-2 in cervical SCC patients underwent NACT before surgery. For the 34 patients who underwent chemotherapy before surgery, 5 cases were with complete remissions, 19 cases were with partial remissions. And the effective rate of chemotherapy was 70.83% (24/34). The expression of miR-143 before and after NACT was analyzed by real time PCR in 24 patients who were treated effectively. The same tissues without chemotherapy were used as control. Expression of miR-143 was up-regulated after NACT treatment, with the relative expression level of 1.35 (0.44 - 4.29). However, there was no statistical significance in expression level of miR-143 before and after NACT (Z = −0.763, P = 0.446) (Figure 3).

**Figure 4:**
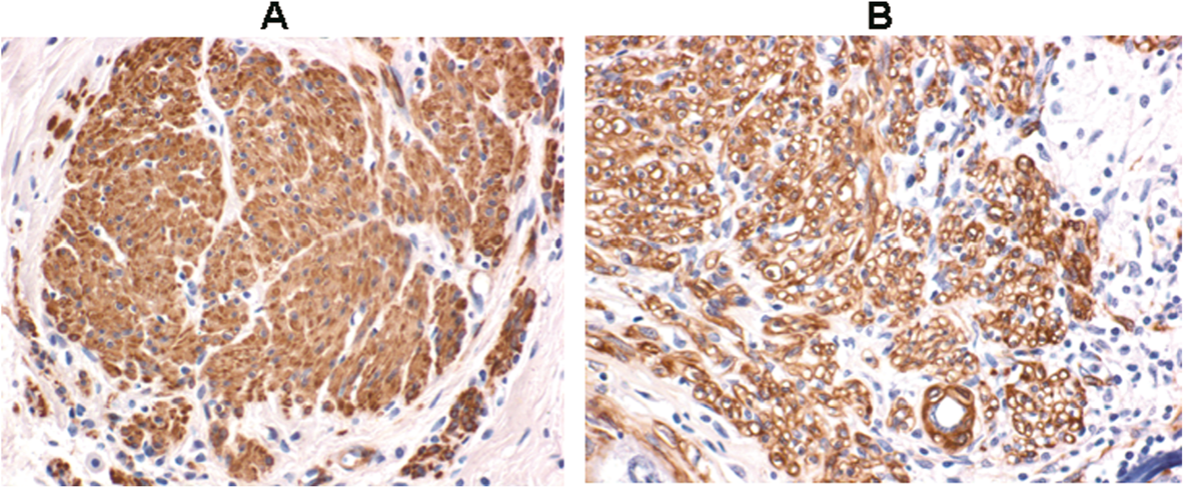
**Bcl-2 expression analysis in cervical SCC tissues before and after neoadjuvant chemotherapy (NACT).** Cervical SCC tissues were collected from 24 cervical SCC patients with effective NACT. Bcl-2 expression before and after NACT was analyzed by immunohistochemistry. Representative immunohistochemical results were shown. Positive staining cells were stained brown-yellow or yellow in the cytoplasm or cell membrane. **(A)** Positive expression of bcl-2 in the cytoplasm of cervical tissues before NACT. **(B)** Positive expression of bcl-2 in the cell membrane of cervical tissues after NACT.

The expression of bcl-2 protein before and after NACT was also analyzed in effective patients with immunohistochemistry. Representative immunohistochemical results were shown in Figure 4. Figure 4A and B showed the positive expression of bcl-2 in fresh cervical tissues before NACT treatment and after NACT treatment. Bcl-2 positive cells were stained brown-yellow or yellow in the cytoplasm or cell membrane. Bcl-2 positive cells were counted and positive expression rate of bcl-2 was calculated. A percentage of bcl-2 positive cells > 10% was defined as positive bcl-2 expression. Statistically, the positive expression rate of bcl-2 was 75% (18/24) before NACT and 54.17% (13/24) after NACT, however, the difference was not statistically significant (χ^2^ = 2.277, P = 0.131) (data not shown).

Thus miR-143 and bcl-2 expression was not significantly changed before and after NACT therapy, suggesting that they may not participate in the Taxol sensitivity response.

## Discussion

Some studies indicate that miR-143 is up-regulated in tumor tissues [10,11]. Also, through inhibition of FNDC3B, miR-143 promotes the metastasis of hepatic carcinoma (animal study) to the lung and the metastasis of prostate cancer cells [12,13]. However, most of the studies demonstrate that miR-143 is down-regulated in solid tumor, including esophageal cancer, bladder cancer and prostate cancer, etc. [14-16]. The target genes of miR-143 include KRAS, MACC1 and bcl-2, etc. [17-19], which are involved in the signaling pathways of ERK5 and MAPK, etc. [20,21]. Among those target genes, bcl-2 is an oncogene involved in cell apoptosis. The function of bcl-2 protein is to inhibit apoptosis by providing cell resistance to multiple apoptosis-stimulating factors. It is reported that through targeting bcl-2, miR-181d inhibited neuroglia cell proliferation and promoted apoptosis [22] while miR-15b and miR-16 played a role in the drug resistance of gastric cancer [23].

Our results indicate that miR-143 was significantly down-regulated in cervical SCC, which was consistent with previous reports [24,25]. They both found that miR-143 expression was down-regulated in cervical cancer cell lines and tissues. However, the relationship between miR-143 expression and clinical pathological features of cervical cancer is rarely studied. In this study, we also analyzed whether miR-143 expression was related with clinical pathological features of cervical cancer. Our results showed that the expression of miR-143 was related to the lymph node metastasis of cervical SCC. Expression of miR-143 was significantly lower in patients with lymph node metastasis than that in patients without lymph node metastasis. Similar results were found in esophageal cancer [26], suggesting that down-regulation of miR-143 might promote the lymph node metastasis of cancer cells. The ROC curve analysis showed that the sensitivity and specificity of miR-143 to evaluate lymph node metastasis were 75% and 60%, respectively. This data suggest that miR-143 may be used as an indicator for lymph node metastasis evaluation in clinic, especially as a reference before surgery. Moreover, our results showed that miR-143 and tumor diameter was negatively correlated. A previous study also showed that the expression of miR-143 in the colorectal cancer tissue with tumor diameter > 5 cm was lower than that with tumor diameter < 5 cm [27]. The sensitivity and specificity of miR-143 to evaluate tumor diameter were 70.5% and 61%, respectively. The self-shedding of exogenous cervical tumor and the underdetermined tumor diameter of endogenous cervical would lower the clinical staging of tumor and influence the individualized treatment of patients. Ivanov et al. reported that in cervical cancer at stage Ib2-IIa, the lymph node metastasis rate and distant metastasis rate were increased as the tumor size enlarged [28], and there was certain correlation between tumor metastasis and tumor size. In addition, we found that the expression of miR-143 was irrelevant to the age, clinical stage, pathological grade and LVSI of the SCC patients.

The infection rate of high-risk HPV in cervical cancer can be up to 50%-90% [29]. In this study, there were 60 cases (78%) of HPV positive cervical SCC patients, in which there were 32 HPV16 positive cases (42%). In comparison with the HPV negative cervical SCC patients, miR-143 was significantly down regulated in the HPV16 positive cervical SCC patients, suggesting that this down regulation might be related with the carcinogenic processes of E6 and E7 genes of HPV16. Researches found that there were 53 miRNAs, including miR-143, which were located at the gene fragile sites and near to the HPV integration sites. Of the HPV integration sites, HPV16 integration site was the most important one [30]. The integration of HPV oncogene to the host cell might influence the formation of miR-143 precursor. In addition, E6 gene of HPV16 could inactivate the tumor suppressor p53, which positively regulated the expression of miR-143 by regulating the synthesis of mature miRNAs [31]. It was also reported that E6 gene of HPV16 could down-regulate the expression of miR-128 [32].

The traditional treatment for cervical cancer is surgery and radiotherapy. However, the relapse rate is relatively high and there are many complications in these methods. NACT can enhance the efficacy of surgery and radiotherapy, control the lesions of cervical cancer, and reduce the lymph node metastasis rate, parametrial infiltration rate and vascular invasion rate [6]. As previously reported, miRNAs are involved in drug resistance of cancer cells. For example, six miRNAs, including miR-21-5p, miR-20a-5p, miR-103a-3p, miR-106b-5p, miR-143-5p, and miR-215, might be able to predict which patients benefit from adjuvant chemotherapy [7]. The miR-143 increased the sensitivity of human colorectal cancer cells to 5-fluorouracil and the sensitivity of prostate cancer cells to docetaxel [8,9]. To determine whether miR-143 is sensitive to Taxol treatment in cervical cancer, the changes in the expression of miR-143 and its target protein bcl-2 before and after NACT were detected in 24 patients who were treated effectively. Results showed that there was no significant difference in the expression of miR-143 in cervical cancer tissues after effective chemotherapy, suggesting that miR-143 was not involved in the effective response of cervical cancer tissue to Taxol. In the 24 effective chemotherapy cases, there was no significant decline of the bcl-2 positive expression after chemotherapy, suggesting that bcl-2, was not involved in the response of cervical cancer tissue to Taxol either. Taxol is able to stabilize the tubulin, inhibit the cell division and proliferation, and arrest the cancer cells at G2 and M phases, thus exerting its anticancer effect. The disturbance of the cell apoptosis pathway is beneficial to the tumor growth and invasion. Under the stimulation of cell growth factors, Rb is phosphorylated by the G1 phase protein cyclin D, and releases E2F, which promotes the gene transcription and progression of cell cycle. Studies indicated that miR-143 was one of the regulating genes of cyclin D1 [33]. In cervical cancer, miR-143 may promote apoptosis and inhibit tumor formation by targeting Bcl-2 [25]. Bcl-2 regulates the activation of p53 gene, and prevents the cell from arresting at the G1 phase [34]. Thus miR-143 could regulate cell cycles directly and indirectly to inhibit cancer cell growth, but may not act synergetically with Taxol. The exact relationship between miR-143 expression and cervical cancer sensitivity to Taxol still needs further investigation.

Cervical cancer is widely recognized to be caused primarily by persistent infection with high-risk HPV. However, HPV infection is not sufficient for cervical carcinogenesis and additional genetic events are involved in cervical carcinogenesis. It is reported that the human telomerase gene (hTERC) is involved in the progression of uterine cervical dysplasia to invasive cancer and can be used for the detection of high-grade cervical intraepithelial neoplasia (CIN) [35,36]. Han et al. reported that immunoreactivities of decreased D2-40 and increased p16^INK4A^ were correlated with higher grade of CIN [37]. Imura et al. found that Laminin-5 was a useful biomarker in the evaluation of invasiveness in cervical adenocarcinoma [38]. In this study, we analyzed the expression of miR-143, the correlation of miR-143 expression with clinical pathological features of cervical cancer, and the sensitivity of miR-143 to Taxol treatment. However, the role of miR-143 expression in evaluating the grade and invasiveness of cervical cancer is not studied and still needs further investigation.

In conclusion, the down regulation of miR-143 was a promoting factor for the occurrence of cervical SCC, and related to the infection of HPV16. The down regulation of miR-143 was also related with tumor size and lymph node metastasis. However, miR-143 was not involved in the effective responses of cervical SCC cells to Taxol.

## Competing interests

The authors declare that they have no competing interests.

## Authors’ contributions

CYX carried out the quantitative real-time PCR, participated in HPV genotype detection, performed the statistical analysis and drafted the manuscript. ZW, CZF and ML carried out the immunohistochemistry assays. MCL conceived of the study, and participated in its design and coordination and helped to draft the manuscript. All authors read and approved the final manuscript.
